# Marine Algae Metabolites as Promising Therapeutics for the Prevention and Treatment of HIV/AIDS

**DOI:** 10.3390/metabo9050087

**Published:** 2019-05-02

**Authors:** Natalya N. Besednova, Tatyana N. Zvyagintseva, Tatyana A. Kuznetsova, Ilona D. Makarenkova, Tatyana P. Smolina, Ludmila N. Fedyanina, Sergey P. Kryzhanovsky, Tatyana S. Zaporozhets

**Affiliations:** 1 Federal State Budgetary Scientific Institution, Somov Research Institute of Epidemiology and Microbiology, Sel’skaya Street, 1, 690087 Vladivostok, Russia; besednoff_lev@mail.ru (N.N.B.); takuznets@mail.ru (T.A.K.); tsmol@mail.ru (T.P.S.); niiem_vl@mail.ru (T.S.Z.); 2 G.B. Elyakov Pacific Institute of Bioorganic Chemistry, Far Eastern Branch of the Russian Academy of Sciences, Pr. 100-letiya Vladivostoka, 159, 690022 Vladivostok, Russia; zvyag@piboc.dvo.ru; 3 Far Eastern Federal University, School of Biomedicine, Bldg. M25 FEFU Campus, Ajax Bay, Russky Isl., 690922 Vladivostok, Russia; fedyanina52@mail.ru; 4 Pacific State Medical University, Ostryakova Ave, 2, 690002 Vladivostok, Russia; kryzhanovskii.sp@dvfu.ru

**Keywords:** marine algae (red, brown, green) metabolites, sulfated polysaccharides, lectins, laminarans, polyphenols, HIV/AIDS, anti-HIV activity

## Abstract

This review presents an analysis of works devoted to the anti-human immunodeficiency virus (HIV) activity of algae metabolites—sulfated polysaccharides (fucoidans, carrageenans), lectins, laminarans, and polyphenols. Despite the presence of a significant number of antiretroviral drugs, the development of new therapeutic and prophylactic agents against this infection remains very urgent problem. This is due to the variability of HIV, the absence of an animal model (except monkeys) and natural immunity to this virus and the toxicity of therapeutic agents and their high cost. In this regard, the need for new therapeutic approaches and broad-spectrum drugs, which in addition to antiviral effects can have anti-inflammatory, antioxidant, and immunomodulatory effects, and to which the minimum resistance of HIV strains would be formed. These requirements meet the biologically active substances of marine algae. The results of experimental and clinical studies conducted in vitro and in vivo are presented, and the issues of the anti-HIV activity of these compounds are considered depending on their structural features. On the whole, the presented data prove the high efficiency of seaweed metabolites and justify the possibility of their use as a potential basis for the development of new drugs with a wide spectrum of activity.

## 1. Introduction

The human immunodeficiency virus (HIV) infection is one of the most dangerous, progressive diseases affecting masses of people. By mid-2017, 36.9 million people globally were living with HIV. In 2017, there were 1.8 million new cases of this disease. Since the beginning of the epidemy, 77.3 million people have become infected with HIV and 35.4 million of them have died from AIDS-related diseases [1,2].

The number of HIV-infected in the world is growing exponentially. The increasing prevalence of HIV transmission during heterosexual intercourse indicates the transition of HIV from the group of drug users to the general population, and involves women in the epidemic process. The vast majority of people infected with HIV live in low- and middle-income countries, and more than 25 million people live in African countries.

According to Pokrovsky et al. [3], it is necessary to prevent losses in the most able-bodied part of the population by introducing more effective programs for the prevention and treatment of HIV/AIDS, since 2% of people aged 35–39 years and 1% of people aged 40–44 are infected with this virus.

In spite of the presence of a significant number of antiretroviral drugs, the development of new therapeutic and prophylactic agents to control this infection remains an extremely urgent problem due to HIV variability, the lack of an animal model (except monkeys) and the natural immunity to this virus, and toxicity of therapeutic agents and their high cost. In this regard, the need for new therapeutic approaches and broad-spectrum drugs, biologically active substances from algae polysaccharides, lectins, phlorotannins) suit these requirements. The results of the experimental report and clinical studies conducted in vitro and in vivo by worldwide scientists prove the high efficacy of these compounds and justify the need to use them as a potential basis for the creation of new broad-spectrum drugs.

## 2. Infectious Agent

HIV infection is caused by a virus belonging to the subfamily of retroviruses, which is called lentiviruses or “slow” viruses. This means that from the moment of infection to the appearance of the first signs of the disease and even more so to the development of AIDS (the last stage of HIV infection), a long period of time, sometimes several years, passes. Currently, HIV is the most studied of all viruses [4]. More than 200 thousand scientific articles devoted to HIV have been published.

HIV exists and multiplies only in the humans, affecting the immune system, eventually depriving it of its ability to resist not only the reproduction of the pathogen, but also to the development of other diseases and infections (tuberculosis, tumors and fungal diseases, severe acute respiratory infection) taking an irreversible course and leading to a lethal outcome.

There are two types of HIV—HIV-1 and HIV-2, differing in antigenic composition. The most common type of HIV-1, is the one that is found everywhere, while HIV-2 is mainly recorded in the African continent.

The basis of HIV supercapsid is a bilayer phospholipid membrane, that surrounds the nucleus containing RNA and enzymes—reverse transcriptase (revertase), integrase, protease. The outer layer of the virus includes structural proteins—glycoproteins—gp 160, 120, 41 kD. The inner layer of the virus covering the nucleus is also represented by proteins—p17, 24, 55. The HIV genome is represented by two identical single-stranded positive viral RNA molecules, each of which consists of 9700 nucleotides. Viruses contain three structural genes:

- gag—group specific antigen—encodes the synthesis of viral proteins of the inner layer;
- pol—polymerasae—encodes the enzymes reverse transcriptase (revertase), protease, integrase and ribonuclease;
- env—envelope—encodes the synthesis of the glycoprotein of the outer layer of gp160, which is further split into gp120 and gp41.

HIV also contain seven regulatory genes that encode the synthesis of more than 20 virus-specific proteins.

The reverse transcriptase catalyzes the synthesis of virus-specific DNA on the viral RNA matrix. Then the viral RNA is converted into DNA and the virus uses the host cell’s machinery to replicate itself during a process called reverse transcription. Ribonuclease promotes to cleavage of the initial RNA, followed by synthesis of the second DNA strand on the first matrix. Endonuclease (integrase) initiates the integration process—the embedding to the cell nucleus DNA copy of viral RNA.

The life cycle of HIV, like other viruses, is made up of several stages. The first of these is the penetration of the virus into the cell with the help of gp120 protein, which is able to interact with the receptor—the CD4 protein on the surface of T-lymphocyte—the target of HIV. This receptor is present on many human cells, but most of all on CD4 –T cells (T-helpers).

During the initial binding of gp120 to CD4, the viral protein changes its shape and binds to the other cell surface proteins—CXCR4 and CCR5, after which another viral protein, gp41, is immersed in the cell membrane. Thereafter, the cell membrane and the virus are merged, and the hereditary material of the virus enters the cell cytoplasm.

The process of reverse transcription occurs, after the penetration of HIV genetic material into the cell, i.e., the transfer of information from viral RNA to DNA. The RNA of the virus using reverse transcriptase begins to synthesize the DNA that is inserted into the genetic apparatus of the cell, having the form of a provirus. It can be kept in an inactive state for a lifetime. Basically, the virus begins to multiply in dividing T-lymphocytes, although it can function in a non-dividing cell. This is the main reason why HIV infection is incurable. When the provirus is activated in the infected cell, there is an intensive accumulation of new viral particles that leads to the destruction of cells and the defeat of new ones. RNA is very unstable when compared to DNA thus, HIV has a tremendous mutation rate—tens of thousands of times faster than for humans.

After the reverse transcription, the virus genome is inserted into the human genome by the viral protein integrase. In HIV infections, those T-cell clones that are specific for HIV proteins are activated first.

After the copy of the virus in the cell genome begins to act, gp41 and gp120 proteins appear on its surface and the rest of the viral proteins and viral RNA appear in the cytoplasm. After some time, new and new copies of HIV begin to bud off the infected cell, eventually destroying the cell membrane. In addition, viral Vpu protein causes increase of permeability of cell membrane. Viral proteins disrupt the cell balance of pro- and anti-apoptotic proteins. If the cells did not die, then, they are actively destroyed by T-killers, which is the standard body’s response to infection with any viruses.

Antibodies that should often prevent the development of infection, in the case of HIV, on the contrary, strengthen it.

Due to the fact that gp120 appears on the surface of the infected cell, the viral particle may likely merge with other CD4-containing cells form a huge multi-core cell (syncytium) that cannot perform any functions but is doomed to death. When an infected cell on the membrane which gp120 is present meets a healthy cell with CD4 receptors, the gp120 CD4 bind and gp41 protein activates to lead the fusion of the membranes of these cells, and then the cytoplasm of these cells is mixed. The formed syncytium retains the ability to further join new healthy cells and merge with them (oligocythemic immunosuppression) for several days, until its death. 

## 3. Anti-HIV Therapy

In world practice, the following groups of antiretroviral drugs are used:

- *Nucleoside*. Nucleoside analogs replacing natural pyrimidine and purine nucleosides, disrupting the synthesis of proviral DNA and suppressing viral replication) and *non-nucleoside* (directly linked to reverse transcriptase near the nucleoside binding site; as a result of complexing with the drugs, this site have an impact and the enzyme binds to a smaller number nucleosides, which significantly slows down the conversion of RNA to DNA);
- *Protease inhibitors*. Suppression of protease activity leads to the formation of immature viral particles, which is unable to infect new cells;
- *Fusion inhibitors*. By specifically binding to the gp41 of HIV-1 outside the cell, the drug blocks the penetration of the virus into the target cell and the fusion of the outer membrane of the virus with the cell membrane;
- *CCR5 receptor inhibitors*. Prevent penetration of HIV to the target cell by acting on the CCR5 co-receptor;
- *Virus integrase inhibitors*. Block the virus enzyme that is involved in the insertion of proviral DNA into the genome of the target cell. Integrase inhibitors affect one of the stages of the process of inserting proviral DNA—the transfer of a DNA strand. After the transfer of the pre-integration complex (proviral DNA in association with integrase) from the cytoplasm to the cell nucleus, integrase joins the cell DNA, which leads to irreversible binding of the proviral DNA and the DNA of the infected cell.

Modern advances in the treatment of HIV infection have been achieved with the help of preparations obtained by traditional methods of chemical synthesis of active molecules that block the action of HIV enzymes or prevent the penetration of the virus into CD4 lymphocytes. The disadvantages of this treatment are that it should be carried out for a lifetime, although, the drugs are very expensive, their use leads to the accumulation of toxic side effects.

In this regard, scientists continue to search for new low- or non-toxic compounds that are effective in HIV infection due to the fact that new therapeutic approaches and broad-spectrum drugs are now needed aimed at various viruses and different stages of HIV replication. They can become potential anti-HIV drugs and minimize the development of resistant viruses [5]. Primarily, such requirements correspond with the components of seaweed (red, brown and green)—sulfated polysaccharides (carrageenans, fucoidans, ulvans), lectins, phlorathannins, and metabolites [6,7]. The current review presents the main results of studies on the anti-HIV effects of individual groups of algal metabolites with various chemical structures presented in the scientific literature to date.

## 4. Marine Algae Metabolites 

### 4.1. Lectins

Lectins of red, brown, and green algae [8], and cyanobacterias such as cyanovirin-N, microvirin, microcrystalline viridis-lectin Scytovirin, Oscillatoria agardhii-lectin, and Griffithsin are considered as potential candidates for preventing sexual transmission of HIV [9] They do not only inhibit the infection of cells with HIV, but can effectively prevent the transmission of the pathogen from infected cells to uninfected CD4^+^ T lymphocytes [6]. Lectins are proteins that bind to certain carbohydrate structures, but do not possess enzymatic activity [7,10]. Due to its exceptional property of glycan recognition, algal lectins are candidates for inhibition of pathogens of various viral diseases [8]. Many lectins inhibit viral replication by interacting with viral envelope glycoproteins [11].

Currently much attention is giving to the development of drugs (they are called microbicides) for the prevention and treatment of HIV infection, and sexually transmitted by persons who practice anal sex. The risk of HIV transmission during anal intercourse without a condom is high, at 138 per 10,000 populations [12]. The use of microbicides causes undesirable side effects, puts the need for scientists to research new compounds for creating such drugs. Currently, extensive research is underway of a new rectal microbicide Griffithsin with MW 12,7 kDa, which was extracted by water from red algae *Griffithsia* sp., collected off the coast of New Zealand [13,14]. 

This lectin binds terminal mannose residues in Man_5-9_5GlcNAc_2_ structures associated with asparagine (N), which constitute the overwhelming majority of N-linked glycans in the layer of HIV-1 type [15]. Griffithsin blocks the binding of gp120 virus glycoprotein to the corresponding cell receptors (gp120, gp41, and gp160), which allows it to neutralize a wide range of laboratory-adapted strains and clinical isolates of HIV-1 and HIV-2 [16]. Griffitsin is considered to be the most potent inhibitor of HIV penetration to date [13,17,18,19]. Griffithsin showed its safety on various cell lines, including the cervical canal cells (End1/E6E7, Ect1/E6E7, CaSki), fibroblasts (3E3) and dendritic (moDc) cell lines [18,19,20,21]. In addition, griffithsin did not give undesirable side effects on the model of vaginal irritation in rabbits [19,22], and was characterized by minimal toxicity in experiments on acute and chronic toxicity [15]. The effectiveness of the griffithsin-based gel in experiments on rhesus monkeys is also described by Girard et al. [16]. The authors found that the gel does not cause any pathological changes in the mucosa of the rectum, as well as in the structure of the microbiota and in the proteome of the mucosa of this part of the intestine. The lectin was able to prevent HIV infection in the tissues of the human cervix without production or with negligible production of pro-inflammatory cytokines [18,20].

The pharmacokinetic profile of this lectin allows for various methods of its application. Thus, the authors recommend to patients self-administer it subcutaneously, and for post-exposure prophylaxis of HIV infection, the authors recommend first intravenous administration to quickly create the necessary concentration of the lectin in the serum. In addition, it is possible oral use of griffithsin to prevent rectal transmission of HIV. All this makes the griffithsin an effective base for drugs, since lectins have an anti-inflammatory activity too [23].

### 4.2. Sulfated Polysaccharides (SPSs)

Sulfated polysaccharides are widely distributed not only in algae, but also in mammals and invertebrates [24]. These compounds represent an extensive class of biopolymers, the content and structure of which vary depending on the type of algae, the place of its growth, climatic conditions, harvest season, method of extraction and many other factors [25].

According to the literature data, SPSs of various origins (fucoidans, galactofucan, dextran sulfate, carrageenans, sulfated chitosans, synthetic and polyvinyl- or polietilensulfaty) possess antiviral activity to many virus pathogens—influenza, hepatitis C, tick-borne encephalitis virus, Newcastle disease, hemorrhagic fever with renal syndrome, Dengue fever et al. [26].

It is known that in the humans the most common heteropolysaccharides are glycosaminoglycans—negatively charged long unbranched polymeric polysaccharides consisting of repeating units—disaccharides. The binding of glycosamines with different ligands leads to post-translational modifications that ensure cell migration, their proliferation, differentiation, etc. Among glycosaminoglycans, the class of heparan/heparansulfates present in the basement membranes, in the extracellular matrix, as well as on the surface of cells in membranes that are able to specifically interact with macromolecules of the extracellular matrix (fibronectin, laminin), enzymes are the main class of heparanbinding molecules (growth factors, chemokines). Glycosaminoglycan mimetics, including heparan/heparan sulfates provide a wide spectrum of biological effects and modulate the effect of many signaling molecules on the cell by binding to other molecules [27].

Natural mimetics of heparansulfates are algae SPSs. Fucoidans and carrageenans can imitate the action of endogenous factors and regulate the functions of micro-organism through key cell and enzyme receptors. Because of this, SPSs have the ability to bind to various receptors on the surface of the host cell and compete with influenza viruses for glycoprotein receptors.

In the last decade, a lot of work has appeared that presents the effectiveness of fucoidans—SPSs of brown algae and carrageenans—SPSs of red algae in HIV infection. The first information about the inhibitory effect of HIV was published in 1987 [28]. These biopolymers exhibit pronounced anti-HIV activity by inhibiting the attachment of viruses to target cell molecules on their membranes. Viral binding peptides are highly conserved regions in a rather variable framework of surface glycoproteins of viruses. They can change a little when antigenic drift, however, they cannot cause resistance of the virus to them. In this regard, SPSs aimed at these target peptides are attractive candidates for the development of antiviral drugs in general and anti-HIV drugs in particular [29,30,31].

The structure of SPSs from brown algae is highly diverse and depends on the type of algae, its reproductive status and other abiotic factors. In fact, each new polysaccharide isolated from algae is a new substance, in the molecule of which there are unique structural elements.

#### 4.2.1. Carrageenans

**Figure 1:**
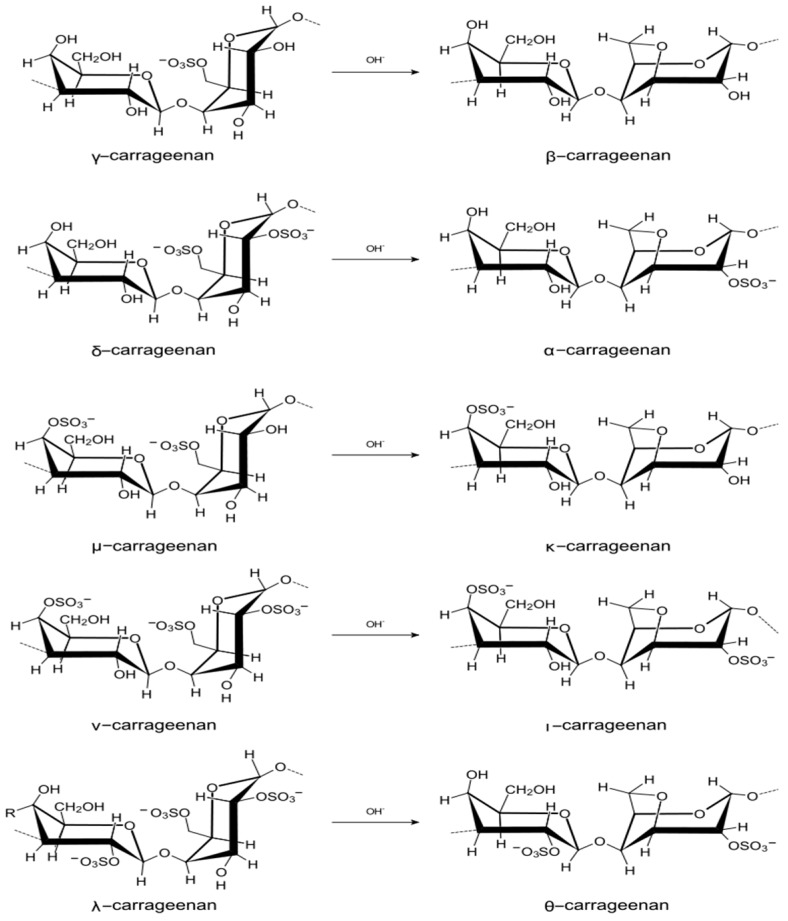
Structural fragments of carrageenans.

Carrageenans are sulphated polysaccharides of red algae whose chemical structure is based on a disaccharide repeating unit consisting of D-galactose residues connected by alternating ß-1→4- and α-1→3-glycosidic bonds [32] (Figure 1) The structural diversity of carrageenans is due to the presence of ß-(l→4)-linked residue of 3,6-anhydrogalactose, as well as the number and location of the sulfate groups in the monosaccharide residues. Regular polysaccharides, the polymer chain which is constructed from repeating disaccharide units of the same type, have received their own name. Natural carrageenans rarely correspond to regular structure, more often they contain repeating units of several types and are represented by an irregular or hybrid structure, which is explained by the multi-stage biosynthesis of polysaccharides in the algal cell wall.

The variability of the primary structure of carrageenans determines the diversity of their macromolecular organization and determines the presence of a wide spectrum of their biological activity [32]. The uniqueness of these hydrocolloids lies in alternating galactose and 3,6-anhydrogalactose residues, which are connected by α-1→3 and β-1→4-glycosidic bonds. A characteristic feature of carrageenan molecules is the presence of a large number of sulfate groups [33]. The number and location of sulfuric acid residues determine the type, form, and function of carrageenans, each of which is actively used in the meat, dairy and confectionery industry to improve the microtexture of products, as a gelling agent, emulsifier and thickener [34].

Among polysaccharides of algae, carrageenans are the most studied in terms of toxicity, pyrogenicity and allergenicity. The safety of their use in food and medical purposes has been confirmed by numerous studies [27].

Among the diverse biological properties of SPSs, antiviral, anticoagulant, immunomodulatory, antitumor, anti-ulcer activity is currently attracting the greatest interest. Sulfated polysaccharides interact with a variety of eukaryotic cell proteins and have a multidirectional effect on the immune response, both inhibitory and stimulating, which allows us to consider carrageenans as possible immunomodulators. It is assumed that the immunomodulatory effect of carrageenans is initiated by α-Gal-(1→3)-Gal epitopes [35]. There is a lot of data on the antioxidant activity of algae polysaccharides [32]. Carrageenans are selective inhibitors of enveloped and unenveloped viruses, and they act primarily by inhibiting adhesion or internalization of the virus into host cells.

The inhibitory effect of polyanionic substances on viruses was established more than four decades ago. However, these observations did not arouse considerable interest among scientists, since such effects were considered nonspecific. In 1987, Nakashima et al. [28] reported on HIV reverse transcriptase inhibition by sulfated polysaccharide from the red alga *Schizymenia pacifica*. Recently, a study was conducted on the anti-HIV activity of a mixture of carrageenans (Sigma) from red algae: the source of k-carrageenan was *Euchema cottoni* alga, x-carrageenan—two algae—*Gigartina aciculaire* and *G. pistillata*. It was found that the antiviral activity of polysaccharides was different, but both were strong selective inhibitors of HIV-1 replication in Human T-cell leukemia (MT4) cells at a concentration of from 0.1 to 0.01 µg/mL, without showing toxic properties to host cells to a concentration of 2.5 mg/mL. Carrageenans not only inhibited the cytopathogenic effect of HIV, but also prevented the HIV-induced syncytium (giant cells) formation. Moreover, the antiviral activity increased with an increase in the molecular weight of the compounds and the degree of their sulfatation. The authors also showed that SPSs can act synergistically with different anti-HIV drugs, including azidothymidine [36,37].

SPSs slowly lead to the formation of HIV resistance, and it is also active against mutants, that become resistant to reverse transcriptase inhibitors. Sulfated galactans from red algae *Grateloupia filicina* (GFP) and *G. longifolia* (GLP) as vaginal preparations for use in HIV infection have been proposed by Wang et al. [38]. The sulfate content of the preparations was 25.7% and 18.5%, respectively. Sulfates were located at C2 for GFP and at C2 and C6 for GLP. Both compounds showed very strong activity against HIV-1 when added simultaneously with the infection of human peripheral blood mononuclear cells and within 2 hours post-infection (EC50s—0.010–0.003 μM, EC90s—0.87–0.33 μM) and present low cytotoxicity.

Regarding the mechanism of carrageenans action in HIV infection, Witvrouw and De Clercq [36] suggest that polysulfates have anti-HIV activity by screening positively charged sites in the V3 loop of the viral enveloped glycoprotein (gp120). The V3 loop is required to attach the virus to heparansulfate of the cell surface, the primary binding site, before more specific binding to the CD4 receptor on CD4^+^ lymphocytes occurs. This is a general mechanism of antiviral activity of polysulfates in relation to enveloped viruses, which makes the possibility to use these compounds not only for treatment, but also for the prevention of viral infections.

The study was carried out in South Africa in 2004–2007 and encompassed more than 6000 female volunteers. It was found that the gel is safe, but not effective, which was confirmed by the results of an in vitro study. In this case, carrageenan was active against HIV at a concentration 100 times higher than when inhibiting, for example, papillomaviruses [33]. In 2008, clinical study of carrageenan-based gel was allowed to establish its effectiveness as a means of blocking sexual HIV infection in women [39]. 

#### 4.2.2. Fucoidans

Fucoidans are sulfated heterogeneous polysaccharides from brown algae, which are mixtures of structurally diverse polysaccharides with different variations of monosaccharide residues (mainly L-fucopyranoses, often galactose and other monosaccharides), types of glycosidic bonds and non-carbohydrate substituents. A detailed structural analysis of fucoidans is currently difficult, so the structural diversity of these polysaccharides is far from being fully investigated [40].

**Figure 2:**
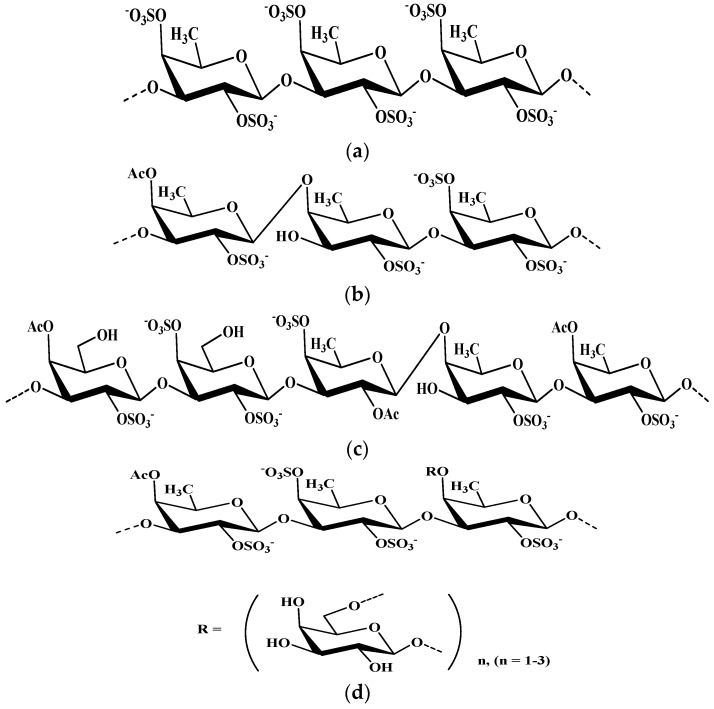
Structural fragments of fucoidans. (**a**) I structural group. (**b**) II structural group. (**c**,**d**) III structural group.

Analysis of the literature data shows that most of the known fucoidans belong to three structural types [41] (Figure 2). The first type contains (1→3)-linked L-fucopyranose residues in the main chain; the second type is alternating (1→3)- and (1→4)-linked residues of L-fucopyranose; the third type of fucoidans (galactofucans) contains fucose and galactose residues, sometimes these monosaccharides are represented in the structures of fucoidans in comparable amounts. In addition to fucose, fucoidans often contain small amounts of other monosaccharides. Fucoidans are rarely found with the main chain represented by uronofucans. It is important that, unlike semi-synthetic, natural SPSs do not exhibit anticoagulant activity at therapeutic doses.

Yet in 1988, Baba et al. [37] found that fucoidan from the brown alga *Fucus vesiculosus* inhibits HIV in vitro and is an azidothymidine synergist. Apparently, this activity was the result of a direct interaction of the polysaccharide with the HIV binding site on human target cells [42].

Dinesh et al. [43] investigated the anti-HIV activity of three SPSs (CFF, FF1, and FF2) from the brown alga *Sargassum swartzii*. The total sugar content in the FF1 and FF2 fractions was 61.8% and 65.9%; the sulfate content—19.2% and 24.5%, uronic acid—17.6% and 13.4%, respectively. The main sugar was fucose (>50%) in both fractions, then galactose. Mannose and xylose were present in small amount, MW of monosaccharides were 45 and 30 kDa, respectively. The authors established the inhibitory effect of the polysaccharides on the p24 antigen, which appears approximately 2 weeks postinfection and allows early diagnosis of HIV infection. The highest inhibitory activity against the p24 antigen (95.6 ± 1.1%) was shown by the polysaccharide FF2 at a dose of 25 μg/mL. The inhibitory activity of azitothymidine taken in the experiments as a positive control was 97.2 ± 1.1% at a dose of 10 μM.

Reverse transcriptase (RT) converts a single-stranded viral RNA genome into proviral DNA, which is an essential stage before its integration into the host’s genomic DNA. The same fraction of FF2 inhibited RT most significantly (78.9 ± 1.43% at a dose of 25 μg/mL) compared to others. The same indicator for azidothymidine was 95.5 ± 1.3%. All fractions have no cytotoxic effects on cells up to concentrations of 1000 μg/mL. The authors associate a higher activity of the FF2 fraction, isolated by anion-exchange chromatography, with higher sulfate content and recommend it as a potential candidate drug against HIV.

Meiyu et al. [44] investigated potential targets for marine sulfated poly-mannoguluronate (SPMG) associated with inhibition of HIV penetration. Results indicated that binding of SPMG either to soluble oligomeric rgp120 or to complexed rgp120-sCD4 mainly resided in V3 loop region. SPMG was shown to be less accessible for sCD4 when sCD4 had pre-interacted with rgp120, though SPMG per se multivalently bound to sCD4 with relatively low affinity. While the pre-incubation of SPMG with rgp120 caused a partial blockade of rgp120 binding to sCD4, suggesting that SPMG either shared common binding sites on gp120 with sCD4 or masked the docking sites of gp120 for sCD4. V3 domain was demonstrated to be the major site mediating interaction of SPMG with complexed rgp120-sCD4. It seems likely that SPMG binds to both rgp120 and sCD4, but has less accessibility for sCD4 when sCD4 has already bound to rgp120. Nevertheless, addition of SPMG either prior to or after the interaction of rgp120 with sCD4 may suppress rgp120 binding to sCD4.

We have already mentioned that the antiviral effect of SPSs, according to scientists [45], is related to the number and location of sulfate groups in their structure. The other results were presented by Thuy et al. [46] which obtained SPSs and their polysaccharides derivatives from three types of brown algae of the genus Sargassum: *Sargassum polycystum* (FSP), *S. moolueri* (FSM) and *Turbinaria ornata* (FTO). These polysaccharides consisted mainly of highly sulfated galactofucans, which is typical for algae of this family. The galactose content was: for FSP—13.70 mol%; for FSM—20.10 mol%; for FTO—20.10 mol%. The monosaccharide composition (except galactose) was presented: for FSP xyl—6.20; gluc—trace; man—10.70 mol%; for FSM xyl—2.60; gluc—1.10; man—1.90 mol%; for FTO: xyl, gluc, man—trace; and fucose content was: for FSM—40.00; for FSP—20.30; for FTO—30.30 mol%. Anti-HIV activity of these polysaccharides were studied on U373-CD4-CXCR4 cells infected with pseudo-viral particles labeled with luciferase. 

The results indicated that all fucoidans showed similar anti-HIV activity with IC_50_ in the range of 0.33 to 0.7 μg/mL. The least toxic was the polysaccharide FSM (20 µg/mL), the other two polysaccharides were more toxic (2 µg/mL). The highest sulfate content was in FSM compared to the other two SPSs, but their antiviral activities were not significantly different. The activity was also not affected by the location of sulfate groups in the structures of fucoidan. Fucoidans inhibited HIV infection if they were preincubated with the virus, but not with the target cells, i.e., fucoidans blocked the early stage of HIV penetration into target cells, screening the positively charged amino acids of the gp120 envelope [47] by durable interaction with structural fragments sulfated in a certain way.

Brown algae contain two types of acid polysaccharides presented in the extracellular matrix—fucoidan and alginic acid. Queiros et al. [48] isolated and characterized fucoidans from several species of brown algae—*Dictyota mertensii*, *Lobophora variegata*, *Spatoglossum schroedery* and *F. vesiculosis*. The polysaccharides from *D. mertensii* were heterofucans containing, predominantly, fucose, galactose, glucose, xylose and/or uronic acid residues. The polysacchride from *F. vesiculosis* was fucan containing only sulfated fucose residues. It was found that fucans at a concentration of 0.5–1.0 μg/mL had a pronounced inhibitory effect on HIV reverse transcriptase. The exception was xylogalactofucan isolated from *S. schroedery*, which did not show inhibitory activity against reverse transcriptase. Alginic acid at a dose of 1 µg/mL inhibited the reverse transcriptase activity by 51.1%. The inhibitory effect of fucans disappeared after their desulfation. After decarboxylation, only xylyofucoglucuronan from *S. schroedery* lost its activity. The results of this study indicate the significance of the sulfate and carboxyl groups in the manifestation of the activity of these polysaccharides.

Trinchero et al. [49] showed that galactofucan fractions from brown algae *Adenocystis utricularis* were active against HIV-1 in vitro. Two of the 5 fractions had a strong inhibitory effect on the replication of HIV-1 in low doses (IC_50_ 0.6 and 0.9 μg/mL, respectively). It was proved that the inhibitory effect is not due to inactivation of the virus, but by blocking the early stages of virus replication, and therefore the authors recommend these substances as good candidates for the creation of preventive and therapeutic drugs against HIV infection.

M.M. Prokofjeva et al. [41] suggest that all natural fucoidans can be considered as potential anti-HIV agents, regardless of the structure of their carbohydrate chain and the degree of sulfation, since their activity occurs at low concentrations (0.001–0.05 µg/mL). In this work, attention was drawn to the significant advantages of algal polysaccharides: the relatively low cost of production, the absence of a pronounced cytotoxic effect, good solubility, and the low degree of the formation of drug resistance of viruses to SPSs with prolonged use.

The authors investigated the anti-HIV activity of various structural types of fucoidans:

- SCF—fully sulfated α-L-fucan from the brown alga *Saccharina cichorioides*—the polysaccharide chain is built mainly from (1→3)-linked α-L-fucopyranose residues;
- FcF—α-L-fucan from the brown alga *Fucus evanescens*—the main chain consists of alternating (1→3) and (1→4) -linked residues of α-L-fucose;
- GF—SgGF, AoGF, SiGF galactofucans—from *Saccharina gurjanovae, Alaria ochotensis, Saccharina japonica* algae, respectively.

The antiviral activity of fucoidans (α-L-fucans and galactofucans) was studied using two model viral systems based on a lentiviral vectors and a replication competent Moloney murine leukemia virus (Mo-MuLV). The investigated fucoidans at the concentration range of 0.001–100 µg/mL have no cytotoxic effects on Jurkat and SC-1cell lines [50].

Lentiviral transduction of target cells to pseudo-HIV particles leads to the expression of a marker gene that induces their fluorescence. At the same time, transduced cells can be detected using flow cytofluorometry. Anti-HIV substances that inhibit the life cycle of HIV-1 prevent the appearance of fluorescent cells in the population. The authors obtained and studied particles containing HIV-1gp120 + gp41 envelope protein.

The results showed that all studied fucoidans demonstrated significant inhibition of transduction of Jurkat cells with pseudo-HIV-1 particles with HIV-1gp120 + gp41 envelope protein in a concentration of 0.001 µg/mL. In these experiments, high molecular weight fucan ScF and galactofucan SjGF showed higher activity with IC_50_ of 0.01 and 0.001 μg/mL, respectively. High-sulfated fucans and galactofucans compared with others showed a higher inhibitory antiviral activity at a concentration of 10 μg/mL.

With regard to the dependence of antiviral activity on the degree of acetylation, the authors found that acetylated galactofucan SjGF showed the highest activity. However, according to the authors, to a greater extent than acetylation, sulfatation, and other structural features, the magnitude of the molecular weight affects the antiviral activity of fucoidans.

In this work, another important, experimentally proven conclusion was made: the antiviral effect of fucoidans is specific for retroviruses using heparan sulfate as primary viral receptors.

### 4.3. Laminarans 

(laminarins) is one of the most common polysaccharides, which is considered a reserve substance of brown algae. These are soluble linear polysaccharides containing 20–30 glucose residues. Mannitol is located at the reducing end of many laminarans. Laminarans, as a rule, have several side branches from glucose, laminaribiosis, or longer β-1,3-linked oligosaccharide fragments attached to the main chain by β-1,6 bonds [51]. Laminarans have a high antiviral activity and low toxicity in vivo [52].

Muto and colleagues [34] reported that laminaran extracted from kelp are proficient to prevent the activity of HIV by preventing the adsorption of HIV on human-derived lymphocytes and the ability of HIV reverse transcriptase, which play an important role for the virus proliferation. This study suggested that laminaran polysaccharides are effective inhibitors on HIV replication and proliferation.

### 4.4. Alginic Acid (Sopolymer of Manuronic and Guluronic Acids)

In China, polysaccharide “911” was obtained from alginate. This polysaccharide exhibited promising activity against HIV-1 at both chronic infection of H9 cells and acute infection of MT4 cells (Human cutaneous T-lymphocyte) in vitro and in vivo. These special effects revealed that “911” drug inhibited the viral replication of HIV via significantly decrementing the activity of reverse transcriptase (RTase), discontinuing the virus adsorption, and improving the defense mechanisms of the host cells [53].

While drugs based on algae metabolites have not appeared on the pharmaceutical market, a number of scientists [54] propose the concept of dietary prevention of HIV infection. One is based on differences in incidence rates in countries where the population consumes significant amounts of algae and in countries in which these hydrobionts are used less frequently. Thus, in Japan and Korea, the incidence of HIV infection in the past 30 years is extremely low (≤0.1%) compared with the United States (0.6%) [55]. The effect of algae is explained by the fact that these hydrobionts, while in a hostile environment, have developed complex chemical protective compounds against bacteria and viruses. Kubanek et al. [56] and Lane et al. [57] revealed more than 20 different antimicrobial compounds from the surface of 2 macroalgae.

Teas, Irhimeh [54] gave to 11 patients with HIV infection a dry algae powder: 2.5 g *U. pinnatiphida* and 3 g Spirullina per day. When prescribing, they proceeded from the dosages used in Japan, where the average daily consumption of algae is about 5.5 g per day. Prior to testing, none of the patients used seaweed in their diet. All patients had a progressive decrease in the level of CD4 lymphocytes and an increase in viral load. After the course of therapy, an improvement in the quality of the patient’s life was noted (patients tested for 30 questions). CD4 cell count and viral load remained stable for 3 months, in one case viral load decreased. The authors conducted phase 1 and phase 2 (not completely for various reasons) of clinical trials and believe that it is necessary to conduct phase 3 trials with a larger number of patients.

### 4.5. Polyphenols

Polyphenolic compounds consist mainly of phorotannins—products of the polymerization of phloroglucin [52,58]. Almost all phenolic compounds are active metabolites of cellular metabolism and play a significant role in various physiological processes of the macro-organism (respiration, photosynthesis, growth, development, and reproduction) [54]. Phlorotanins have a hepatoprotective and antitumor effect, they are powerful antioxidants. The antioxidant activity of these compounds is 2–10 times higher than that of ascorbic acid and tocopherol [51,59].

These high hydrophilic compounds with a wide range of MW from 126 to 650 kDa are found in red and brown algae [60,61,62,63]. Sea brown algae accumulate various fluorineglucinol-based polyphenols. The polyphenol fraction of brown algae is characterized by the predominant content of phloroglucinol polymers—phlorotannins. The highest content of polyphenolic compounds is characteristic for brown algae [64]. In the dry mass of algae, phloratannins account for about 20% [65]. Polyphenols isolated from seaweed are used as a valuable resource in the manufacture of new herbal medicines that were previously created on the basis of only higher land plants [66].

Phlorotannins are divided into 4 subclasses:

- fugalols and phloretols (phlorotannins with an ether bond);
- fukol (with phenyl bond);
- fucofloretols (with ether and phenyl bond);
- eckols (with dibenzodioxin bond).

Antiviral activity of polyphenols is manifested mainly in relation to enveloped viruses [67]. Ahn et al. [68] and Artan et al. [69] presents the inhibitory effects of florotanins isolated from the alga *Ecklonia cava* Kjelman, on reverse transcriptase, integrase and HIV protease.

Bce yтoчнить So, according to Ahn et al. [68] for 8.8′-dieckol IC_50_ was 0.51 μM with respect to reverse transcriptase, 81.5 μM—against protease; for 8.4′′′-dieckol—5.31 μM to reverse transcriptase, 36.9 μM to protease; for diphlorethohydroxycarmalol 9.1 μM to reverse transcriptase, 25.2 μM to integrase; for 6.6″-dieckol—1.07 μM to reverse transcriptase. These data suggest that all phorotanins effectively inhibit reverse transcriptase. Separate compounds with less efficiency inhibit integrase and protease. Attention is drawn to the fact that inhibition of reverse transcriptase by 8.8′-dieckol was 10 times higher than inhibition by 8.4′-dieckol, although these florotanins are eckol dimers. In this regard, the authors consider 8.8′-dieckol as a new non-nucleoside HIV-1 inhibitor.

Similar results were obtained by Artan et al. [69] and patented [70]. The authors isolated and characterized 6.6′-dieckol, which inhibited the formation of syncytium under the HIV action (EC_50_—1.72 μM), the lytic effect (EC_50_—1.23 μM), as well as the production of p24 virus antigen (EC_50_—1.26 μM). In addition, the compound selectively inhibited the activity of reverse transcriptase (EC_50_—1.07 µM) and the penetration of HIV into cells. Unlike other tannins 6.6′-dieckol did not show cytotoxicity at concentrations that almost completely inhibited the replication of HIV-1. Another drug of this group—8.4′′′-dieckol is also highly appreciated by Karadeniz et al. [71].

An interesting substance for further research as an anti-HIV-1 agent, gallic acid (GA), was described by Zhang et al. [72]. The authors obtained *Phyllanthus urinaria* algae extract with a high content of polyphenols. The toxicity of the extract and one of its components, gallic acid was investigated on the culture of human T-lymphocytes (MT-4). Both the extract and GA showed anti-HIV activity with an IC_50_ of 0.61 µg/mL and 0.76 µg/mL, respectively. It was also shown that the extract and GA interact with HIV-1 RT, gp120 and p24. GA was rapidly absorbed and slowly removed from the body of rats after oral administration of this compound; it was found in the lungs, kidneys, heart, and spleen of rats. Also, it is known that GA is a strong antioxidant [73].

These data indicate the promise studies of florotanins potential for medicine application against HIV infection. 

## 5. Conclusions

In the literature of the last decade, there are many works devoted to the anti-HIV activity of substances isolated from algae. The formation of resistance of HIV pathogens to drugs emerging on the pharmaceutical market requires new approaches to the treatment of this disease. It is necessary to have drugs with different mechanisms of action in the arsenal of drugs, as well as those that, in addition to antiviral effects, can have anti-inflammatory, antioxidant, and immunomodulatory effects, and to which the minimum resistance of HIV strains would be formed.

As presented in this review, various algae metabolites (sulfated polysaccharides, lectins, phenolic compounds, etc.) have such properties. Unfortunately, there are currently no drugs on the pharmaceutical market for treating and preventing HIV infection based on these promising, non-toxic compounds with not only antiviral, but also anti-inflammatory, antioxidant, immunomodulatory, and other beneficial properties. Basically, this is due to the difficulties of their standardization, since the structural study of these biopolymers is complicated by a large variety depending on many factors (algae species, place of growth, method of isolation, degree of purification).

At the same time, scientists received from native polysaccharides of brown algae chemically pure, structurally characterized and homogeneous samples SPSs with low molecular weight [74]. SPSs are agonists of cell receptors of innate and adaptive immunity, have antioxidant, anti-bacterial, and anti-inflammatory properties and, most importantly, prevent the penetration of HIV into the human cells, and inhibit the activity of viral enzymes and replication of virus particles. These unique substances present perspectives as the basis for development of natural products or novel functional ingredients and potential drugs with a wide spectrum of action.

The main undesirable side effect of SPSs could be their anticoagulant activity. However, oral and parenteral application of these compounds in therapeutic doses is quite safe. In addition, the relatively low cost, significant yield of the final product, high antiviral activity, the almost complete absence of toxicity and the formation of pathogens resistance, good solubility, significant reserves of natural sources and the possibility of cultivation of algae make SPSs promising candidates for creating drugs with antiviral, including, anti-HIV targeting. Algae lectins and phloratannins also have high anti-HIV potential. In recent years, these compounds also attracted great attention of scientists.

The authors hope that this review will motivate researchers interested in developing drugs for HIV infection based on natural biologically active substances. Extensive studies are needed on the effectiveness of algal metabolites, not only in vitro, but also in vivo, as well as the synergistic activity of these biopolymers.
